# Experimental observation of spin Hall effect of light using compact weak measurements

**DOI:** 10.1515/nanoph-2024-0217

**Published:** 2024-07-11

**Authors:** Jeonghoon Choi, Sangmin Shim, Yeseul Kim, Peng Tang, Guoqiang Li, Junsuk Rho, Dasol Lee, Minkyung Kim

**Affiliations:** School of Mechanical Engineering, 65419 Gwangju Institute of Science and Technology (GIST), Gwangju 61005, Republic of Korea; Department of Biomedical Engineering, Yonsei University, Wonju 26493, Republic of Korea; Department of Mechanical Engineering, Pohang University of Science and Technology (POSTECH), Pohang 37673, Republic of Korea; International Joint Research Laboratory of New Energy Materials and Devices of Henan Province, School of Physics and Electronics, Henan University, Kaifeng 475004, P.R. China; Department of Chemical Engineering, Pohang University of Science and Technology (POSTECH), Pohang 37673, Republic of Korea; Department of Electrical Engineering, Pohang University of Science and Technology (POSTECH), Pohang 37673, Republic of Korea; POSCO-POSTECH-RIST Convergence Research Center for Flat Optics and Metaphotonics, Pohang 37673, Republic of Korea

**Keywords:** precision metrology, photonic spin Hall effect, polarization, tilted polarizer, reflection, refraction

## Abstract

The spin Hall effect of light, a phenomenon characterized by the transverse and spin dependent splitting of light at an optical interface, is highly promising for collecting precise quantitative data from interfaces and stands as an appealing option for improving precision metrology. This high level of precision is attributed to the principles of weak measurement. Since its conceptual introduction, the spin Hall effect of light has been empirically observed through weak measurement techniques, adhering closely to the initially proposed experimental configuration. Recently, it has been suggested that the setup can be downsized without compromising precision. Here, the first experimental demonstration of “compact weak measurement” is achieved by observing the spin Hall effect of both reflected and refracted light. Compared to the conventional weak measurement, this compact setup performs the same measurements but requires less free space by replacing the two convex lenses with a set of concave and convex lenses. The compact weak measurement demonstrates excellent agreement with theoretical predictions and experimental findings from traditional setups across both isotropic–isotropic and isotropic–anisotropic interfaces. The experimental validation of the compact configuration paves the way for the practical application of the spin Hall effect of light in devices with a smaller form factor.

## 1 Introduction

**Figure 1:**
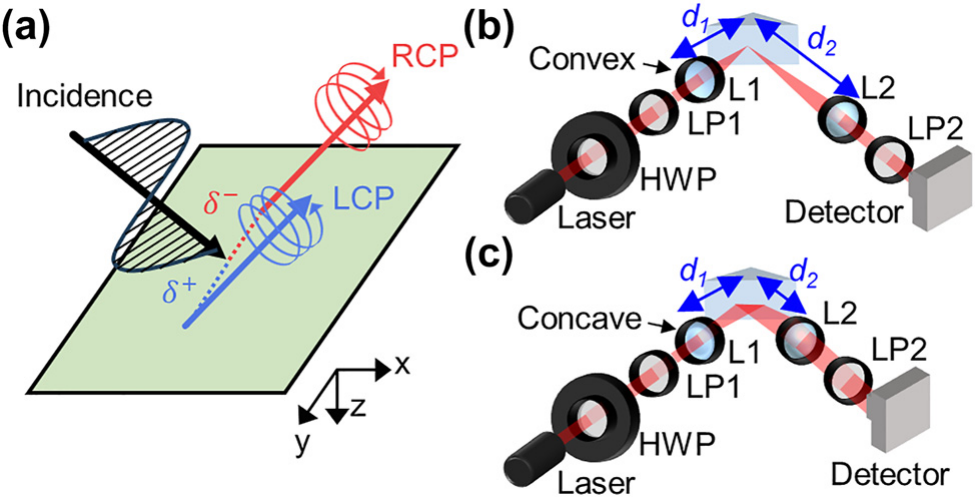
Schematics of SHEL and its setups. (a) Conceptual illustration of SHEL. (b, c) Schematics of (b) conventional and (c) compact weak measurement setup. HWP: half wave plate, LP: linear polarizer, L: lens.

Linearly polarized light refracted or reflected at any optical interface is split into two circularly polarized components oppositely along the transverse direction (Figure 1a) [1], [2], [3], [4], [5]. Analogous to the conventional spin Hall effect of electrons [6], [7], this spin-dependent splitting is termed the spin Hall effect of light (SHEL) and has gained continuous attention for its potential in nanoscale beam splitting [8], [9], [10], [11], [12], [13], [14], [15] and spatial differentiation [16], [17], [18], [19]. Another promising application of the SHEL is a precision measurement of unknown interface parameters. The spin Hall shift, a measure of the transverse displacement, sensitively varies with interface properties and can be precisely quantified up to subnanometer scale [20], rendering the SHEL a valuable tool for precisely determining interface and material properties such as thickness [21], concentration [22], [23], humidity [24], magnetic properties [25], and the number of atomic crystals [26]. Central to the application of SHEL in precision metrology is the concept of weak measurement, an optical technique devised more than a decade ago, inspired by quantum weak measurements [27].

The amplification of the spin Hall shift via weak measurement [28], [29] depends on two factors: the propagation effect through lenses and polarization filtering through linear polarizers [20], [30], [31]. Traditionally, weak measurement involves preselection and postselection parts (Figure 1b), each comprising a lens and a polarizer. Preselection defines the incident polarization and reduces the Rayleigh length to enhance amplification, while postselection adjusts the output polarization and collimates the light. Here, the propagation factor *F* = *d*/*z*_*R*_ is determined, where *z*_*R*_ = *k*_0_ *w*_0_^2^/2 denotes the Rayleigh length, *k*_0_ = 2*π*/*λ* represents the wave number, *λ* is the wavelength, *w*_0_ is the beam waist, and *d* symbolizes the propagation distance from the Gaussian focus to the second lens (L2), corresponding to its focal length (*f*_2_). This factor corresponds to the magnification ratio of the reflected image through L2 and directly relates to the weak measurement’s amplification factor. Thus, the interface experiencing the SHEL is precisely positioned at the focal plane between the two lenses, with distances from the first lens (L1) to the interface and from the interface to L2 being *f*_1_ and *f*_2_, respectively (*d*_1_ = *f*_1_ and *d*_2_ = *f*_2_ in Figure 1b). However, it has been proven recently that this positioning of the interface is not essential but it can be placed anywhere between the preselection and postselection parts [32]. This understanding has led to the theoretical proposal of a compact weak measurement setup, characterized by a shorter propagation distance between the lenses (|*f*_2_| – |*f*_1_| instead of *f*_1_ *+ f*_2_) as depicted in Figure 1c, although experimental validation had been pending.

This article reports the first experimental validation of the compact weak measurement. Experiments are conducted in two configurations: one with light reflected at an isotropic–isotropic interface and another with light refracted at an isotropic–anisotropic interface. The weak signals measured in both setups closely align with theoretical predictions, affirming that the compact configuration delivers reliable outcomes despite its smaller size.

## 2 Results and discussion

### 2.1 Observation of spin Hall effect of reflected light

First of all, the SHEL at the interface of a BK-7 prism, which has a refractive index of 1.515, is observed using the conventional weak measurement setup (Figure 1b). We use the helium–neon laser, characterized by a wavelength of 632.8 nm and a beam diameter of 680 μm, with the incident angle (*θ*_*i*_) set at 45°. The focal lengths of L1 and L2 are set to *f*_1_ = 50 mm and *f*_2_ = 100 mm, respectively. The horizontally polarized light incident on the prism is tightly focused by L1, resulting in a beam waist of approximately 29.6 μm at the prism surface. The spin Hall shift of the reflected light, prior to amplification, can be calculated as follows [33]:

(1) $$\delta_{H}^{\pm }=\mp \frac{\cot\theta_{i}}{k_{0}}\text{Re}\left(1+\frac{r_{s}}{r_{p}}\right),$$

where the subscript *H* denotes horizontally polarized incident light, the superscripts *+* and – indicate the left and right circularly polarized components of the reflected light, respectively, and *r*_*s*_ and *r*_*p*_ represent the Fresnel reflection coefficients for *s* and *p* polarizations, respectively. At this air–prism interface, the spin Hall shift of the reflected light is 225.0 nm (Eq. (1)). This magnitude is considerably less than the pixel size of standard detectors and cannot be observed directly. In contrast, the weak signal (*W*) exhibits a linear relationship with *δ*, as established in ref [20].

**Figure 2:**
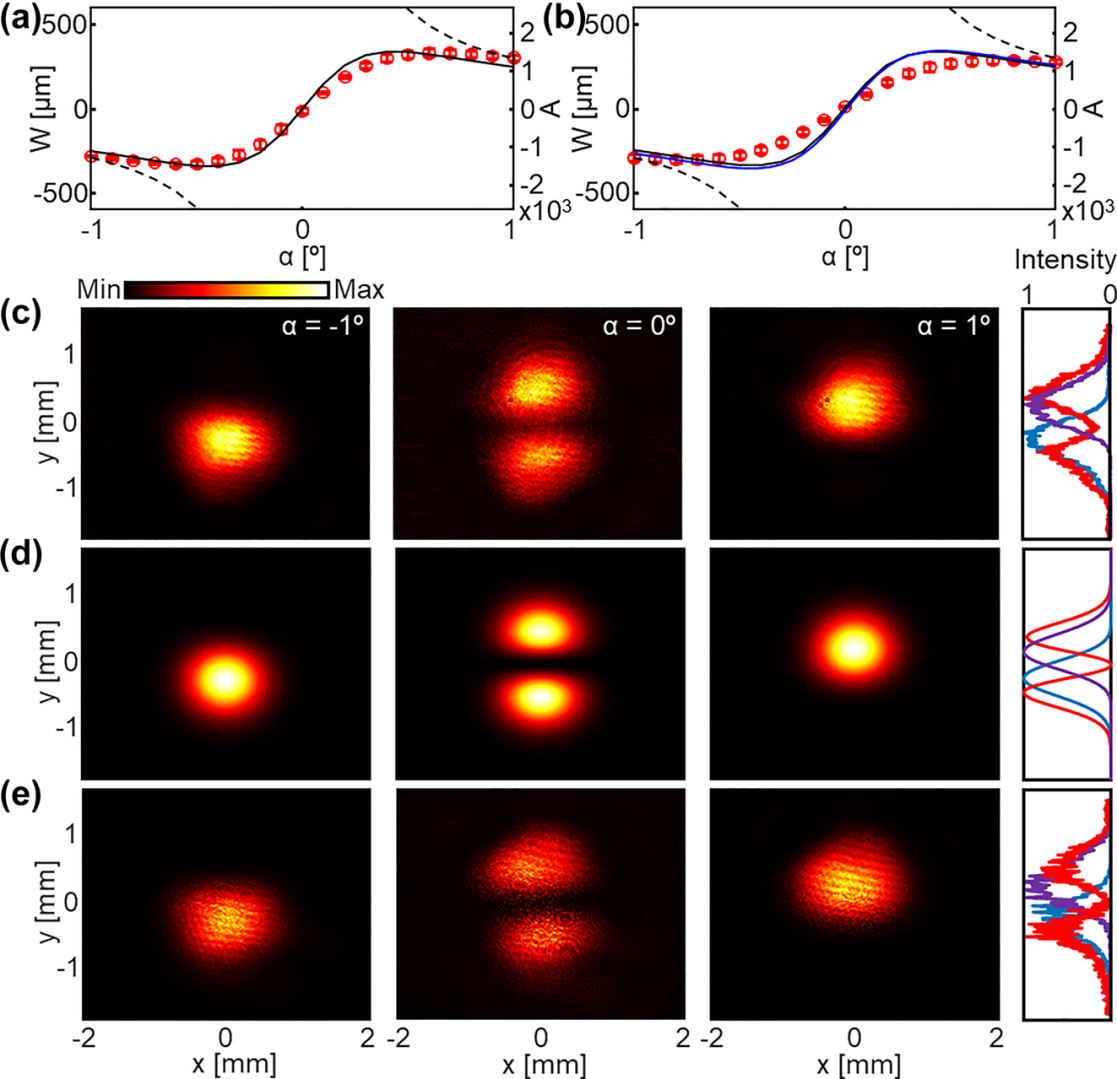
Weak values experimentally measured using the weak measurements. (a, b) Weak values under various postselection angles from −1° to 1° when the incidence is horizontally polarized using (a) conventional and (b) compact weak measurements. Black dashed: approximated theory (Eq. (2)), black solid: theory without the approximation (Eq. (3)), blue: simulated, red markers: measured. (c–e) Intensity distributions of the postselected beam when the postselection angles are −1° (left), 0° (middle), 1° (right). (c) Measured using compact setup, (d) simulated, and (e) measured using conventional setup. One-dimensional intensity profiles along the *y*-axis are shown on the right side. Blue: *α* = −1°, red: *α* = 0°, purple: *α* = 1°.

(2) W=Aδ=Fδ⁡cot⁡α,

where *A* is the amplification factor, *α* is the postselection angle – angular difference between the perpendicular axis of the preselection polarization and postselection polarization. The preselection and postselection states are (1, 0) and (−sin *α*, cos *α*), respectively. In our experiments, *F* = 22.96 and *α* varies from −1° to 1°, resulting in the maximum value of *A* of approximately 1,542. The measured results (Figure 2a, red markers) agree well with the analytic *W* (Eq. (2), dashed curve in Figure 2a) for a large *α* that satisfies

(3) $$\frac{\left|\delta \right|}{w_{0}}\ll \min\left(\tan\alpha ,\cot\alpha \right),$$

but deviate from it for small *α* due to non-negligible higher-order terms. Instead, the results follow the modified weak value reported in ref. [34] (solid curve in Figure 2a). Standard deviations of seven consecutive measurements are denoted as error bars.

Subsequently, we implement the compact weak measurement by substituting L1 with a concave lens possessing identical focal lengths, *f*_1_ = −50 mm, as depicted in Figure 1c. Through this configuration, the incident light diverges upon passing through L1, reflects at the prism surface with a much larger beam diameter, and is then collimated by L2. The lenses are aligned to ensure that the propagation distance from L1 to L2 equals the difference between their focal lengths (*d*_1_ *+ d*_2_ = |*f*_2_| – |*f*_1_| see Figure 1c). At a sufficiently large postselection angle *α* (≈5°), the postselected beam exhibits a similar beam radius to that observed in the conventional weak measurement setup, suggesting that the compact configuration maintains the same *F* value as the conventional approach. The weak signals obtained using the compact weak measurement setup, represented by the red markers in Figure 2b, closely match the theoretical predictions (depicted by the black curve). The standard deviations calculated based on five repeated measurements are indicated as error bars.

This agreement between experimental outcomes and theoretical expectations is attributed to the commutative relationship between reflection and free space propagation, which can be mathematically expressed as

(4) $$\left[\begin{array}{cc} r_{p} & \frac{k_{y}}{k_{0}}\left(r_{p}+r_{s}\right)\cot\theta_{i}\\ -\frac{k_{y}}{k_{0}}\left(r_{p}+r_{s}\right)\cot\theta_{i} & r_{s} \end{array}\right]$$

and exp(*ik*_*z*_ *L*), respectively, where *k*_*y*_ and *k*_*z*_ are the wave numbers along the *y*- and *z*-axes, respectively, and *L* is the propagation distance. Provided that the total phase gain through the preselection and postselection processes remains the same, the location at which the SHEL occurs does not influence the outcomes of the weak measurement (refer to ref. [32] for a detail theoretical proof). An underlying assumption for the applicability of the compact weak measurement setup is the weak-coupling condition (Eq. (3)), wherein theoretical models, irrespective of whether approximations are employed or not, yield similar results (as evidenced by the comparison between the black solid and dashed curves in Figure 2a and b). Thus, we compute the expected values of the weak values in the compact setup numerically using the angular spectrum method (Figure 2b, blue curve) [32]. The agreement between both experimentally measured and simulated results (blue curve) with theory extends beyond the weak-coupling regime, demonstrating the robustness of the methodology.

The two-dimensional intensity distributions of the postselected beam and their cross-sectional profiles along the *y*-axis captured when *α* = −1°, 0°, 1° (Figure 2c) display excellent agreement with both numerically derived distributions (Figure 2d) and experimental data acquired using the conventional method (Figure 2e). The amplified displacement from its original center is observed for *α* = ±1° in opposite direction (compare Figure 2c, left and right). In contrast, significant distortion in the beam profile appears when the preselection and postselection polarization states are orthogonal (*α* = 0°, middle column of Figure 2c). This characteristic two-hump pattern, originating from the transversal nature of light, i.e., momentum and electric field being mutually perpendicular, is a signature of the weak measurement-based amplification of the SHEL [31], [35] and is also observed in our compact setup. Furthermore, while the theoretical proposal of the compact weak measurement is based on the refractive configuration [32], our findings substantiate that the same principles are equally applicable to a reflective configuration, broadening the scope of its utility. The intensity difference in the two experimental data (Figure 2c and e) is attributed to the different input intensities determined by the retardation angle of the half waveplate and the neutral filter and is irrelevant to the SHEL itself or the amplification process.

**Figure 3:**
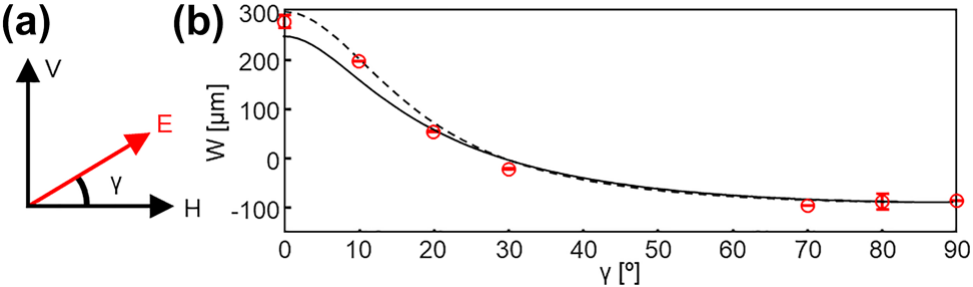
Observation of SHEL under other linear polarizations. (a) Schematic showing the polarization angle and (b) weak values under different polarization angles.

For further investigation, we also measure *W* by varying the incident polarization (Figure 3a). The spin Hall shift of linearly polarized light is expressed by the following equation:

(5) $$\delta_{\gamma }^{\pm }=\delta_{H}^{\pm }\cos^{2}\gamma_{R}+\delta_{V}^{\pm }\sin^{2}\gamma_{R},$$

where *γ* is the polarization angle of the incidence (Figure 3a),

(6) $$\gamma_{R}=\cos^{-1}\frac{r_{p}\cos\gamma }{{\left({\left(r_{p}\cos\gamma \right)}^{2}+{\left(r_{s}\sin\gamma \right)}^{2}\right)}^{1/2}}$$

is the polarization angle of the reflected light, and

(7) $$\delta_{V}^{\pm }=\mp \frac{\cot\theta_{i}}{k_{0}}\text{Re}\left(1+\frac{r_{p}}{r_{s}}\right)$$

is the spin Hall shift of vertically polarized light [33]. The measurements are conducted similarly to the previous (Figure 2) except that the postselection angle is determined relative to the perpendicular axis of *γ*_*R*_, rather than the perpendicular axis of preselection polarization. Accordingly, the preselection and postselection states are defined as (cos *γ*, sin *γ*) and (−sin(*γ*_*R*_ + *α*), cos(*γ*_*R*_ + *α*)), respectively. Given that the spin Hall shift exhibits divergence at the Brewster angle (approximately 56.6°) under horizontal polarization, the analytical predictions of the weak signal using the first-order (black dashed line in Figure 3b, Eq. (2)) and those incorporating higher-orders terms (black solid) show noticeable difference near *γ* = 0° for *α* = 1°. However, this discrepancy diminishes as *γ* increases. The experimental measured weak signals (red markers) exhibit similar tendency with the theoretical expectations, exhibiting a consistent trend across a broad spectrum of *γ*.

### 2.2 Observation of spin Hall effect of refracted light

**Figure 4:**
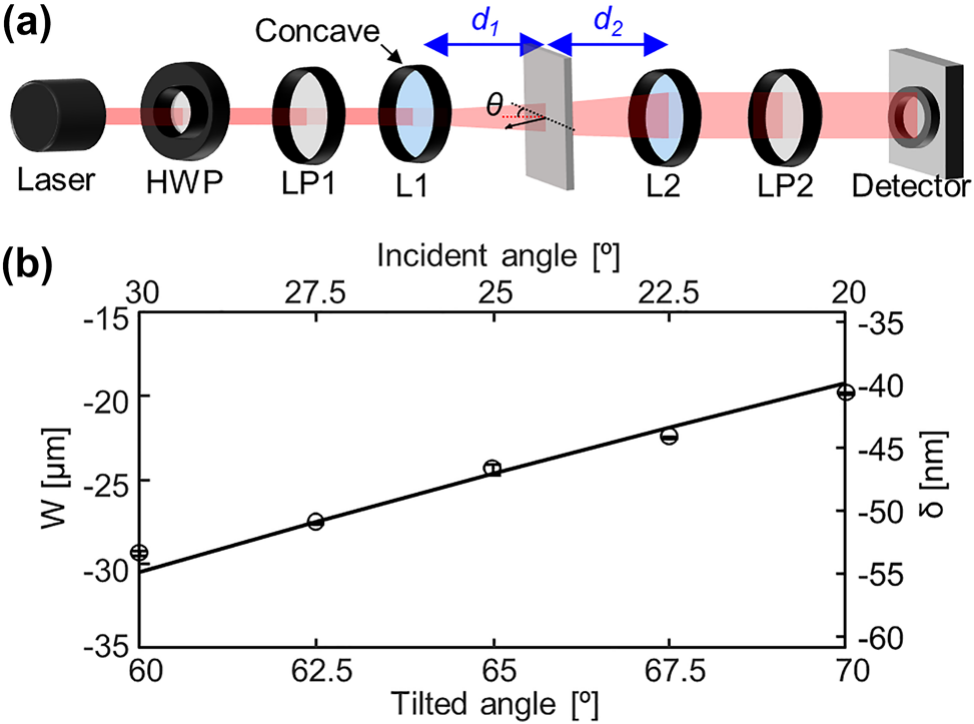
Observation of the spin Hall effect of refracted light through a tilted polarizer. (a) Schematic of a compact weak measurement setup and (b) measured results under vertically polarized incidence. Postselection angle is set as 2.5° in all measurements.

To further prove the validity of the compact weak measurement approach, the spin Hall effect of refracted light through a tilted polarizer is examined (Figure 4a). This SHEL at the anisotropic plate has been regarded as a special case, distinct from that observed at Snell-Fresnel interfaces, and termed geometric SHEL [36]. Later, the SHEL at this isotropic–anisotropic interface has been integrated in the framework of standard SHEL by Bliokh et al. [35]. and known to be amplified using conventional weak measurement setups (a schematic of which is available in Figure 1a of ref. [35]). The compact weak measurement setup to observe SHEL at the tilted polarizer is illustrated in Figure 4a. This tilted polarizer, which transmits one polarization components while completely blocks the other, is a typical example of an anisotropic material and thus light obliquely passing through the tilted polarizer can be understood as light refracted at an isotropic-anisotropic interface. This transmission type setup clearly manifests the advantages of the compact weak measurement, i.e., the reduction in the required free space distance from *f*_1_ *+ f*_2_ to |*f*_2_| – |*f*_1_|, which corresponds to a third of the original propagation distance for our setup parameters. The only difference between this experiment and those conducted at isotropic–isotropic interfaces (Figures 2 and 3) is that the argument of the cotangent function in Eq. (1) should be the tilted angle *θ* = 90 − *θ*_*i*_, the relative angle between the absorption axis of the polarizer (black dashed in Figure 4a) and the light propagation direction (red dashed) [35]. Weak signals for vertically polarized incidence are obtained by varying the tilted angle of the polarizer (Figure 4b). In all measurements, the focal lengths of the lenses remain unchanged (*F* = 22.96) and *α* = 2.5°, resulting in *A* = 525.0. The experimentally obtained weak signals agree well with the theory, confirming the usefulness of the compact weak measurement not only in refractive setups but also in examining SHEL at isotropic−anisotropic interfaces.

## 3 Conclusions

In conclusion, this work presents the first experimental validation of the compact weak measurement by observing the spin Hall effect of both reflected and refracted light. First, the spin Hall effect of reflected light at an interface between two isotropic media is quantified utilizing the compact setup and compared with results from the conventional setup. Subsequently, the spin Hall effect of refracted light through an anisotropic plate is examined, employing identical focal lengths but within a refractive-type arrangement. Across both experimental scenarios, the measured data closely align with theoretical predictions and the results obtained via conventional weak measurement. The successful experimental demonstration of the compact weak measurement highlights its potential to significantly reduce spatial requirements in SHEL-based precision metrology, thereby broadening the spectrum of feasible applications, including the development of miniaturized sensors. This advancement not only validates the compact measurement approach as a viable alternative to traditional methods but also heralds a new phase in the practical application of SHEL for enhanced metrological precision and device miniaturization.

## Supplementary Material

Supplementary Material Details
