# Supplementary material for: Experimental observation of spin Hall effect of light using compact weak measurements
Source: Nanophotonics. 2024 Jul 11;13(20):3877–82. doi: 10.1515/nanoph-2024-0217 (PMC11465994; doi:10.1515/nanoph-2024-0217)
Supplement: Supplementary file 1 — Supplementary Material Details [file j_nanoph-2024-0217_suppl_001.pdf]

GIST | School of Mechanical Engineering

**Minkyung Kim,** Ph.D.
Assistant Professor
Tel: +82-62-715-2772, E-mail: m.kim@gist.ac.kr
123 Cheomdangwagi-ro, Buk-gu, Gwangju 61005, Republic of Korea

June 9, 2024

**Subject: Re-submission of a manuscript –** "Experimental observation of spin Hall effect of light using compact weak measurements" **– by** Jeonghoon Choi, Sangmin Shim, Yeseul Kim, Peng Tang, Guoqiang Li, Junsuk Rho, Dasol Lee, and Minkyung Kim

Dear Prof. Jia Zhu,

Thank you for sending the decision letter with the detailed referees' reports concerning our recent submission to *Nanophotonics*. We have carefully reviewed and revised the manuscript accordingly. We submit here the revised manuscript for your consideration, along with detailed point-to-point responses to the reviewers' comments.

We are very pleased by the depth and quality of the questions raised by the reviewers, which were greatly helpful for the revisions. Both reviewers acknowledged the impact and novelty of our work and provided many constructive and positive queries and suggestions to further improve the completeness of our work. Following the reviewers' feedback, we have revised the manuscript and responded to them faithfully in the response letter. In addition, we have thoroughly proofread the manuscript once more to further enhance its quality.

We believe that the referees' comments have improved the quality and presentation of our manuscript and are confident that our work reporting the first experimental demonstration of the compact weak measurement not only meets the high standard of *Nanophotonics* but will also find a broad readership. We appreciate the editor for giving us the opportunity to strengthen our manuscript with valuable comments and queries and hope the editor will find these revisions satisfactory.

Thank you for your full consideration in advance.

Sincerely,
On behalf of all authors
Minkyung Kim, Ph.D.
Assistant professor
School of mechanical engineering, Gwangju Institute of Science and Technology (GIST)
123 Cheomdangwagi-ro, Buk-gu, Gwangju, Republic of Korea
m.kim@gist.ac.kr

*Minkyung Kim*
